# Author Response to Denteneer et al

**DOI:** 10.1093/ptj/pzaa040

**Published:** 2020-03-23

**Authors:** Max Jakobsson, Annelie Gutke, Lidwine B Mokkink, Rob Smeets, Mari Lundberg

We thank Denteneer et al for taking the time to read our systematic review of the level of evidence for reliability, validity, and responsiveness of physical capacity tasks (or “physical functioning tests,” as Denteneer et al refer to them).^1^ We agree that the research field of identifying high-quality physical capacity tasks is a very important one, particularly to avoid biased and imprecise results in research and clinical practice. Additionally, physical capacity tasks appear to be a valuable complement to back-specific patient-reported outcome measures (PROMs), such as the Roland-Morris Disability Questionnaire, as they measure a different aspect of the patient’s status. ^2–4^ Denteneer et al pose a few questions in their letter, and we address these below.

Regarding the question of the generalizability of our systematic review, we used the inclusion criteria of at least 6 weeks’ pain duration to both include patients with subacute (6–12 weeks duration)^5^ and chronic LBP (more than 12 weeks duration^5^ to enable data synthesis for both subgroups. However, as stated in the results section, the majority of the patients had chronic LBP and not subacute LBP. We, therefore, did not consider it appropriate to generalize the results to patients with subacute LBP. Consequently, we stated that 5-Repetition Sit-To-Stand, 50-Foot Walk, 5-Minute Walk, Progressive Isoinertial Lifting Evaluation (PILE), Timed “Up&Go,” and 1-Minute Stair Climbing tasks were promising physical capacity tasks for patients with chronic LBP and not subacute LBP. As we argue in the discussion section, we consider the results to be generalizable to most patients with chronic LBP who adhere to the eligibility criteria of the study. However, as we also describe in the study, a portion of the results are primarily generalizable to patients with back-related diagnoses known to affect walking capacity severely; for example, lumbar spinal stenosis.

We thank Denteneer et al for pointing out the article by Hodselmans et al,^6^ which indeed reports good test-retest reliability for PILE. Due to the heterogeneous terminology used in the literature for describing these types of tests (eg, as described by Harriet Wittink^7^), we used a broad search strategy that identified 7900 potential articles. However, it appears that the article by Hodselmans et al was not identified by our search strategy. We believe that the research field would benefit from a more homogeneous terminology, preferably based on the *International Classification of Functioning, Disability and Health (ICF)*,^8^ to simplify the identification of articles in future systematic reviews.

Regarding the loaded forward reach test, it was excluded due to the eligibility criteria that all tests had to be a measure of capacity under the ICF *activity* domain.^8^ We considered the movement in loaded forward reach (leaning forward as far as possible without losing balance) too artificial to be considered a part of the activity domain. Moreover, the ICF describes “reaching” as “using the hands and arms to extend outwards and *touch and grasp* something,” which is not done in loaded forward reach.^8^ Instead, we consider the loaded forward reach test to include a movement composed of several body functions of the ICF (balance, for example). Nevertheless, the test as such appears to have good test-retest reliability as Denteneer et al write in their letter and also showed in their systematic review.^9^

Finally, in line with the results of our systematic review, Denteneer et al point out that more validity and responsiveness studies of physical capacity tasks are needed. Another insight of our systematic review was that most of the validity and responsiveness studies were only of fair methodological quality, particularly due to the fact that many studies lacked a priori hypotheses for validity and responsiveness. Such hypotheses are essential for investigating validity and responsiveness, as described by the Consensus-Based Standards for the Selection of Health Measurement Instruments (COSMIN) initiative.^10–12^ We hope to see future high-quality studies that investigate validity and responsiveness with the use of such hypotheses.

## Notes

**Max Jakobsson, Annelie Gutke, Lidwine B. Mokkink, Rob Smeets, Mari Lundberg**

M. Jakobsson, RPT, PhD, Back in Motion Research Group, Department of Orthopaedics, Institute of Clinical Sciences, University of Gothenburg, Mölndal Hospital, Göteborgsvägen 31, 431 80 Mölndal, Gothenburg, 41326 Sweden; and Occupational and Physical Therapy, Campus Högsbo, Sahlgrenska University Hospital, Gothenburg, Sweden.

A. Gutke, RPT, PhD, Division of Physiotherapy, Department of Health and Rehabilitation, Institute of Neuroscience and Physiology, University of Gothenburg.

L.B. Mokkink, PhD, Amsterdam UMC, Vrije Universiteit, Department of Epidemiology and Biostatistics, Amsterdam Public Health Research Institute, Amsterdam, the Netherlands.

R. Smeets, MD, PhD, Department of Rehabilitation Medicine, Research School of CAPHRI, Maastricht University, Maastricht, the Netherlands; and CIR Revalidatie, Eindhoven, the Netherlands.

M. Lundberg, RPT, PhD, Division of Physiotherapy, Department of Health and Rehabilitation, Institute of Neuroscience and Physiology, University of Gothenburg; and Division of Physiotherapy, Department of Neurobiology, Care Sciences and Society (NVS), Karolinska Institutet, Stockholm, Sweden.
